# Comparative Profiling of Circulating Exosomal Small RNAs Derived From Peruvian Patients With Tuberculosis and Pulmonary Adenocarcinoma

**DOI:** 10.3389/fcimb.2022.909837

**Published:** 2022-06-30

**Authors:** Heinner Guio, Victor Aliaga-Tobar, Marco Galarza, Oscar Pellon-Cardenas, Silvia Capristano, Henry L. Gomez, Mivael Olivera, Cesar Sanchez, Vinicius Maracaja-Coutinho

**Affiliations:** ^1^ Laboratorio de Referencia Nacional de Biotecnología y Biología Molecular, Instituto Nacional de Salud, Lima, Peru; ^2^ Facultad de Ciencias de la Salud, Universidad de Huanuco, Huánuco, Peru; ^3^ Advanced Center for Chronic Diseases - ACCDiS, Facultad de Ciencias Químicas y Farmacéuticas, Universidad de Chile, Santiago, Chile; ^4^ Centro de Modelamiento Molecular, Biofísica y Bioinformática - CMB, Facultad de Ciencias Químicas y Farmacéuticas, Universidad de Chile, Santiago, Chile; ^5^ Department of Genetics, Human of Genetics Institute of New Jersey, Rutgers University, Piscataway, NJ, United States; ^6^ Departamento de Oncología Medica, Instituto Nacional de Enfermedades Neoplásicas, Lima, Peru; ^7^ Instituto Vandique, João Pessoa, Brazil

**Keywords:** exosomes, circulating RNAs, small RNAs, small RNA sequencing, microRNAs, non-small cell lung cancer, tuberculosis, Peruvian

## Abstract

Tuberculosis (TB) is one of the most fatal infectious diseases, caused by the aerobic bacteria *Mycobacterium tuberculosis*. It is estimated that one-third of the world’s population is infected with the latent (LTB) version of this disease, with only 5-10% of infected individuals developing its active (ATB) form. Pulmonary adenocarcinoma (PA) is the most common and diverse form of primary lung carcinoma. The simultaneous or sequential occurrence of TB and lung cancer in patients has been widely reported and is known to be an issue for diagnosis and surgical treatment. Raising evidence shows that patients cured of TB represent a group at risk for developing PA. In this work, using sRNA-sequencing, we evaluated the expression patterns of circulating small RNAs available in exosomes extracted from blood samples of Peruvian patients affected by latent tuberculosis, active tuberculosis, or pulmonary adenocarcinoma. Differential expression analysis revealed a set of 24 microRNAs perturbed in these diseases, revealing potential biomarker candidates for the Peruvian population. Most of these miRNAs are normally expressed in healthy lung tissue and are potential regulators of different shared and unique KEGG pathways related to cancers, infectious diseases, and immunology.

## Introduction

Tuberculosis (TB) is one of the most fatal infectious diseases, caused by the aerobic bacteria *Mycobacterium tuberculosis* (Dye and Williams, 2010). It is estimated that one-third of the world’s population is infected with the latent (LTB) version of this disease, with only 5-10% of infected individuals developing its active (ATB) form (Bhatt and Salgame, 2007). TB is the leading infectious disease in deaths, overtaking AIDS, with 1.3 million deaths in 2016, according to the World Health Organization (World Health Organization, 2017). Despite the global efforts to control its transmission and the amount of effective treatments available, the number of annual deaths caused by this disease is almost unchanged. Together with the increasing incidence of multidrug resistance, it makes TB a public health issue in both developing and developed countries (Falzon et al., 2015; Maitra et al., 2017; Yang and Ge, 2018). In Peru, the incidence for tuberculosis was 116 cases per 100,000 habitants in 2020 (World Health Organization, 2021).

Pulmonary adenocarcinoma (PA) is the most common and diverse form of primary lung carcinoma (Kerr, 2009). It accounts for 40% of all lung cancers (LC) and is the most prevalent in people who have never smoked (Kerr, 2009; de Groot et al., 2018). In Peru, 20% of deaths between 2003 and 2016 were attributed to cancer related (Kerr, 2009; de Groot et al., 2018) diseases (Zafra-Tanaka et al., 2020). The simultaneous or sequential occurrence of TB and LC in patients has been widely reported (Silva et al., 2013) and is known to be an issue for diagnosis and surgical treatment (Cicėnas and Vencevičius, 2007). Increasing evidence shows that patients cured of TB represent a group at risk for developing PA. It might be the result of changes in bronchial and alveolar mucosa caused by the infectious disease (Cukic, 2017). Additionally, it could be related to changes in immune surveillance for cancer cells in the lungs, resulting from the increased number of regulatory T cells (Tregs) in the micro environment, especially in the tuberculosis related granulomas (Ahmed and Vyakarnam, 2020).

Small RNAs are non-protein coding molecules, smaller than 200 nucleotides in length, comprising a myriad of RNA classes with a diverse set of functional roles (e.g., microRNAs, small nucleolar RNAs, Piwi-interacting RNAs), including a huge number small transcripts without an associated function (Storz, 2002; Maracaja-Coutinho et al., 2019). Expression analyses have shown that these small molecules are spatially and temporally precisely regulated and a perturbation in their gene expression patterns can result in pathological phenotypes, including different types of cancer, as well as neurological and cardiovascular diseases (Maracaja-Coutinho et al., 2019). Among the small RNAs, the microRNA (miRNA) class is the most widely studied. It is well recognized for its participation in post-transcriptional control. miRNAs are highly conserved non-coding RNAs which act by regulating gene expression by base-pairing. Therefore, changes in transcriptional output of these molecules could reflect anomalies in global transcription inside the cells. In addition, miRNAs can be exported and imported by cells in vesicles and protein carriers, therefore, allowing their detection in bodily fluids (Weber et al., 2010). In this context, miRNA profiles can be associated with disease progression or differentiation between diseases of similar symptoms (Weiland et al., 2012; Mori et al., 2019), supporting its importance in clinical cases.

A new trend in molecular biology is the use of fluid samples, such as blood or urine, in disease prognostics and diagnosis in replace of invasive biopsies (Maracaja-Coutinho et al., 2019). These body fluids carry large lipoprotein complexes, such as exosomes or microvesicles (MVs), which encapsulate proteins, along with DNA and RNA molecules and are actively released by living cells (Kishikawa et al., 2015). The genetic material encapsulated in these MVs are stable and protected from degradation and can be used as noninvasive biomarkers for both chronic and infectious diseases (Leung and Wu, 2015; Matamala et al., 2018).

In this work, we applied sRNA-sequencing on exosomes extracted from blood samples of Peruvian patients affected by latent tuberculosis, active tuberculosis, or pulmonary adenocarcinoma in order to evaluate the expression patterns of available circulating small RNAs. A plethora of sRNAs were identified and the differential expression analysis revealed a set of 24 microRNAs perturbed in these diseases, revealing potential biomarker candidates for the Peruvian population. Most of these miRNAs are normally expressed in healthy lung tissue and are potential regulators of different shared and unique KEGG pathways related to cancers, infectious diseases, and immunology.

## Method

### Study Participants and Ethics Statement

The study was approved by the Ethics Committees of the National Institute of Health and the National Institute of Cancer from Peru. Healthy controls (HC; n = 4) and patients (n = 19) signed an informed consent to participate in this study. The age range of participants was 18-70 years old, with both men and women participating in the study (**Supplementary Table 1**). The selection was based on availability of samples and consent from patients, according to the funding available and the timeframe of the project (February 2013 to December 2015). Samples of patients with TB (n = 8) and PA (n = 7) were collected before clinical treatment. LTB patients (n = 4) were included according to the following criteria: (i) tuberculin skin test (TST) > 15mm; (ii) X-rays negative; and (iii) sputum negative to Xpert *Mycobacterium tuberculosis* (MTB)/rifampicin (RIF). ATB (n = 4) patients were included according to the following criteria: (i) TST > 15mm; (ii) X-rays positive; and (iii) sputum positive to bacteria. For PA, the pathological report was necessary for the recruitment of patients. The exclusion criteria were: (i) recurrence (recidivism) or reinfection of TB; (ii) HIV infection or primary immunodeficiency; (iii) evidence prior to the disease of a body mass index <18 Kg/mt2; (iv) hematological disorders; (v) drug addiction; and vi) pregnant women. General characteristics for each subject used in this study can be observed in **Supplementary Table 1**.

### Blood Samples Collection and Exosomes Isolation

A total of five milliliters of peripheral blood were collected from each participant into a sterile resin tube without anticoagulant. Samples were centrifuged at 3500 rpm for 10 min, and the supernatant serum was removed, aliquoted, and stored at -20°C. From this, 250 ul of serum was used for exosome isolation using Total Exosome Isolation Kit (Invitrogen, USA). Exosome characterizations were performed through nanoparticle tracking analysis (NTA) using a NanoSight instrument (Malvern Medical, UK).

### RNA Extraction, Library Preparation and Sequencing

Total RNA isolation was performed from exosomes using Total Exosome RNA & Protein Isolation Kit (Invitrogen). To characterize miRNA expression profiling, small RNA sequencing was conducted by a commercial service (SBI, System Biosciences, USA). According to the manufacturer’s instructions, the RNAs were ligated with 3′ RNA adapters before undergoing ligation with a 5′ adapter. The adapter-ligated RNAs were then subjected to low-cycle reverse transcription RT-PCR. The resulting PCR products were selected according to their length in a PAGE gel, according to the protocol for the TruSeq^®^ Small RNA Sample Prep Kit (Illumina, USA). The purified library products were evaluated using the Agilent 2200 Bioanalyzer and diluted to 10 pM for cluster generation *in situ* on the HiSeq 2500 (Illumina, USA) single-end flow cell, followed by sequencing.

### Bioinformatics Analyses

Raw reads were trimmed using Trimmomatic version 0.36 (Bolger et al., 2014). The first five bases in each extremity were removed when their quality average presented a score below 30. A sliding window quality filtering of four nucleotides was implemented in order to eliminate reads with a window average quality below 30. The reconstruction of the repertoire of small RNA transcripts available in all samples, its annotation, and expression levels estimation were obtained using a similar approach to that from Orell et al. (Orell et al., 2018), Matamala et al. (Matamala et al., 2018) and Sotomayor-Flores et al. (Sotomayor-Flores et al., 2020). Reads smaller than 15 nucleotides after the trimming process were discarded and the resulting clean data was mapped to the human genome GRCh38.p12, obtained from Ensembl database (Cunningham et al., 2019), using Bowtie version 1.0.0 (Langmead, 2010). One mismatch was allowed in the mapping and reads with more than 10 alignments were removed. SAM files from the mapping process were converted into BED format and used for coordinates-based assembly through BEDtools (Quinlan, 2014). In order to estimate the small RNA transcripts expression levels, we recovered the reads belonging to each assembled RNA. Transcriptional reconstructed fragments with a length smaller or equal to 200nt were considered as bona fide small RNAs when it presented at least 50 reads in half of the samples from a particular condition, otherwise it was considered as potential noise and was eliminated.

The functional annotation of each expressed small RNA was obtained by cross-referencing their genomic coordinates against the microRNAs available in miRBase version 21 (Kozomara et al., 2019) and the ncRNAs from Ensembl version 92 (Cunningham et al., 2019), considering an overlap of 70% between the small RNA transcript and the reference RNA. The final expression matrix generated during the assembly process was used for differential expression analysis using DESeq2 version 1.28.0 (Love et al., 2014). Small RNA transcripts were considered as statistically differentially expressed when presenting a *p-value* smaller than 0.05 and a log2 fold-change cutoff of 1.5.

The expression of perturbed circulating miRNAs in lung tissue was obtained from the data available in miRmine (Panwar et al., 2017) database (version 2017). To have an approximation of the possible metabolic pathways regulated by differentially expressed miRNAs, a search for KEGG pathways targeted by validated miRNA-mRNA interactions was carried out using miRPath version 3.0 (Vlachos et al., 2015) and TarBase version 7.0 (Karagkouni, 2015). We used the standard parameters suggested by miRPath to consider a pathway as enriched: p-value and MicroT threshold of 0.05 and 0.8, respectively.

## Results

### Small RNA Expression Profiling of Serum Derived Exosomes in HC, PA, LTB, and ATB Individuals

**Figure 1:**
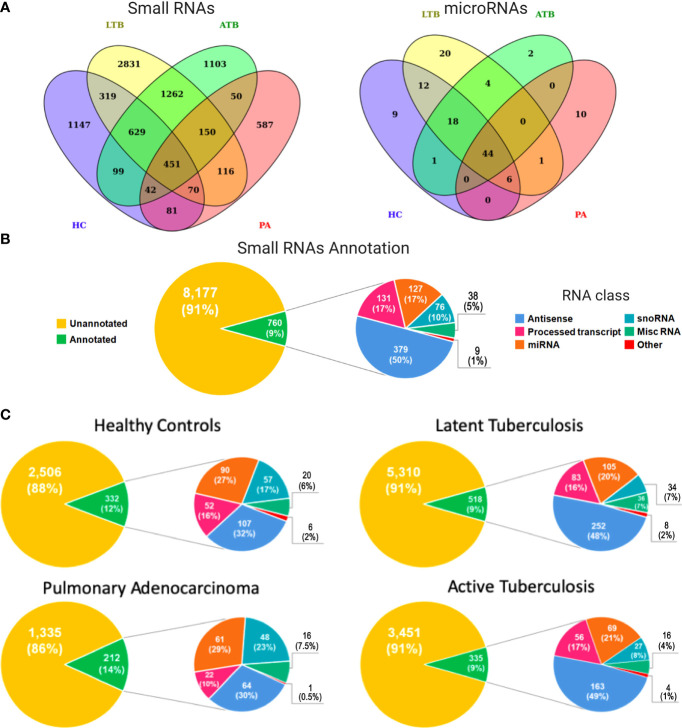
Expression and functional annotation of circulating small RNAs in healthy controls (HC), pulmonary adenocarcinoma (PA), latent tuberculosis (LTB) and active tuberculosis (ATB) individuals. **(A)** Venn diagram containing all small RNAs (left panel) and microRNAs (right panel) in each sample type. **(B)** Pie charts representing the functional annotation of all small RNAs expressed in all studied conditions together. **(C)** Pie charts representing the functional annotation of all small RNAs expressed in each studied condition independently.

We used next generation sequencing (NGS) of exosomes isolated from diseased and healthy Peruvian individuals to obtain a genome-wide expression profiling of circulating small RNAs associated with LTB, ATB, PA, and HC. Considering all analyzed subjects, this screening revealed a total of 8,937 small RNA transcriptional fragments (**Figure 1A**, left panel). Interestingly, the majority of expressed transcripts were identified as condition specific, with 5,668 (63.42%) expressed exclusively in HC, LTB, ATB, or PA individuals. Latent tuberculosis was the condition presenting the higher number of exclusive small RNAs, with 2,831 (31.67%) transcripts expressed specifically in these patients. Healthy controls presented 1,147 (12.83%), active tuberculosis presented 1,103 (12.34%), and pulmonary adenocarcinoma 587 (6.56%). Both tuberculosis conditions (LTB and ATB) shared a total of 2,492 (27.88%) small RNAs, while 1,480 (16.56%) circulating RNAs were identified in patients with cancer and tuberculosis. A total of 787 (8.80%) transcripts were shared between LTB and PA individuals and 693 (7.75%) between ATB and PA (**Figure 1A**).

Our annotation pipeline was able to retrieve 760 (8.50%) circulating RNAs (**Figure 1B**). Of these, 250 (33%) were part of well-characterized small RNA classes, like ribozymes, microRNAs (miRNAs), small nuclear RNAs (snRNAs), small nucleolar RNAs (snoRNAs), and small Cajal body-specific RNA (scaRNA). The annotation revealed a subset 127 miRNAs (**Figure 1A**; right panel), of which 44 (35%) were identified in all analyzed subjects, while 41 (32%) were associated with a specific condition (HC, LTB, ATB or PA). A total of 38 (5%) transcripts were annotated as miscRNA (miscellaneous RNAs), a particular class of short RNAs without functional annotation available in Ensembl database. Based on Ensembl, the other 510 (67%) RNAs were annotated as different sub-classes of long non-coding RNAs yet to be functionally characterized, such as “Processed_transcript” (131, 17%) and “Antisense” (379, 50%). The set of small RNAs annotated as long transcripts could be a byproduct processed in order to develop their function or even a product of degraded long RNAs available inside exosomes.

When considering each condition separately (**Figure 1C**), the potential byproducts of long non-coding RNAs were more representative in both tuberculosis conditions, reaching 48% and 49%, respectively, for LTB and ATB annotated small RNAs. These numbers decreased to 32% and 30% in healthy controls and PA patients, respectively. Interestingly, the proportion of miRNAs and snoRNAs followed an inverse similar pattern, with a higher percentage of miRNAs and snoRNAs expressed in normal (27% for miRNAs; 17% for snoRNAs) and cancerous (29% for miRNAs; 23% for snoRNAs) individuals, compared to LTB (20% for miRNAs; 7% for snoRNAs) and ATB (21% for miRNAs; 8% for snoRNAs) patients. The complete list of annotated small RNAs can be assessed at **Supplementary Table 2**.

### Differentially Expressed Circulating Small RNAs and MicroRNAs in Tuberculosis and Pulmonary Adenocarcinoma

**Figure 2:**
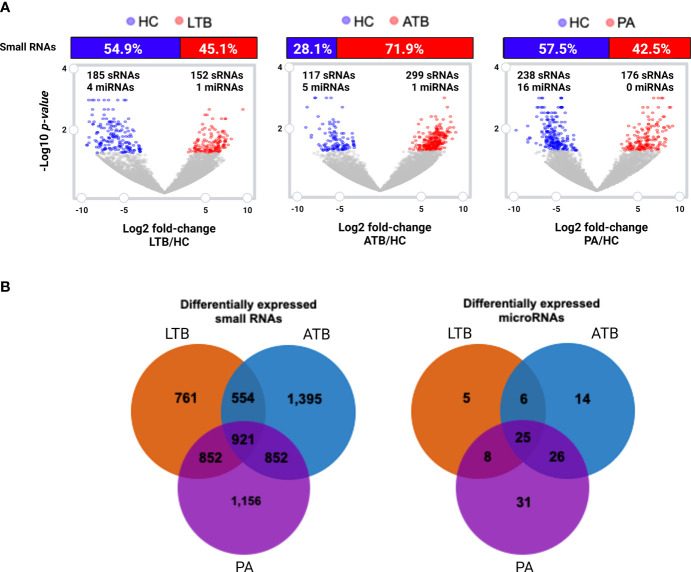
Differentially expressed (log2 fold-change cutoff of 1.5; *p-value* cutoff of 0.05) small RNAs and microRNAs. **(A)** Volcano plot and histogram representing the sets of differentially expressed circulating small RNAs identified in LTB, ATB and PA patients compared to healthy individuals (HC). The number of differentially expressed miRNAs are represented in each figure. **(B)** Venn diagram comparing the set of differentially expressed small RNAs and miRNAs in each disease, compared to healthy individuals.

To gain further insight on the putative relevance of circulating small RNAs to PA, LTB, and ATB, we investigated the set of statistically differentially expressed transcripts compared to healthy controls. Differential expression analysis revealed a total of 899 circulating small RNAs perturbed in at least one of the studied diseases (**Figures 2A, B**), of which 57 appeared as shared within all illnesses (**Figure 2B**). In our analysis, we identified a set of 688 RNAs exclusive to a particular disease, with 157 exclusive to LTB, 230 exclusive to ATB, and 301 exclusive to PA. A total of 113 small RNAs showed to be shared with tuberculosis and pulmonary adenocarcinoma, with 155 presented in both types of tuberculosis, 25 shared only within LTB and PA, and 31 only within ATB and PA. A total of 185 circulating small RNAs appeared as downregulated in LTB, with 152 as upregulated; 117 appeared as downregulated in ATB, with 299 as upregulated; and 238 appeared as downregulated in PA, with 176 as upregulated.

When considering those circulating small RNAs annotated as microRNAs, we identified a total of 24 miRNAs as statistically differentially expressed in at least one of the studied diseases (**Figures 2A, B**; **Supplementary Table 3**). Only one miRNA (miR-125a-5p) was identified as perturbed in all diseases, with another 22 exclusives to each particular disease, and only two (miR-125a-5p and mir-203a) shared within both tuberculosis types. Other three miRNAs (miR-143-3p, miR-210-3p and miR-20a-5p) presented as exclusive to LTB; four miRNAs (hsa-mir-23b, hsa-mir-17, hsa-mir-584 and miR-181b-5p) presented as exclusive to ATB and 15 as unique to PA (**Supplementary Table 3**). The majority of these miRNAs appeared as downregulated in each studied disease, with the exception of miRNA mir-20a-5p and mir-584, which appeared as upregulated in LTB and in ATB, respectively.

**Figure 3:**
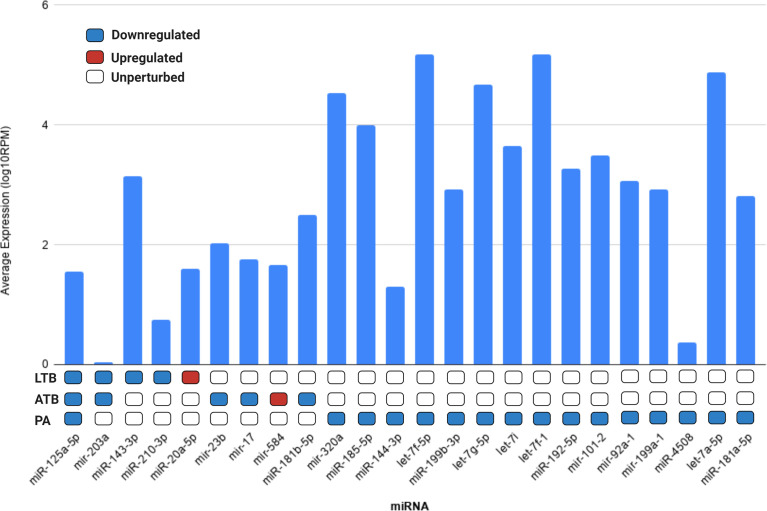
Average expression of each differentially expressed miRNA in lung tissue according to miRMine database (Panwar et al., 2017). Values are displayed in log10 of reads per million (RPM). Blocks colored in dark blue, red, and white represent the miRNAs upregulated, downregulated, or unperturbed in each disease compared to control. The fold-change values for each differentially expressed miRNAs can be accessed in **Supplementary Table 3**.

Next, we evaluated if the set of differentially expressed circulating miRNAs could be derived from the lung tissue, by cross-referencing the expression values of each miRNA with the data available in the miRmine (Panwar et al., 2017) database (version 2017). This repository integrates the expression profiles of miRNAs retrieved and re-analyzed from publicly available normal and diseased human tissues and cell lines RNA sequencing data. Our analysis revealed that most of the differentially expressed miRNAs are highly expressed in the lung, with the exception of mir-203a and miR-4508 (**Figure 3**). The expression changes of each differentially expressed miRNAs can be accessed at **Supplementary Table 3**.

### Pathways Potentially Affected According to Differentially Expressed miRNAs

**Figure 4:**
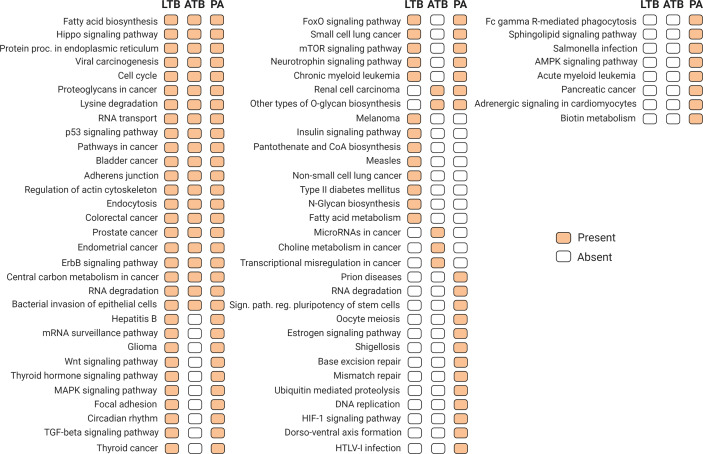
Target KEGG metabolic pathways regulated by differentially expressed miRNAs (p-value and MicroT threshold of 0.05 and 0.8, respectively), according to experimental validated miRNA-mRNA interactions available in miRPath version 3.0 (Vlachos et al., 2015) and TarBase version 7.0 (Karagkouni, 2015). Orange and white blocks indicate presence or absence, respectively, of a particular pathway in each condition.

In order to identify an association of the differentially expressed circulating microRNAs, with metabolic pathways related with the studied diseases, we searched for KEGG pathways targeted by validated miRNA-mRNA interactions according to miRPath (Vlachos et al., 2015). Our results identified miRNAs regulating pathways associated with different infectious diseases and immunological response to infection, including tuberculosis, as well different cancer related metabolic pathways (**Figure 4**).

## Discussion

Exosomes or extracellular vesicles are secreted from many cell types, which can circulate in biological fluids and play important roles in intercellular communication (Bortoluzzi et al., 2017). They carry different molecules like lipids, proteins, nucleic, and ribonucleic acids. Exosomes of patients affected by different diseases can shed light on potential molecules secreted by the cells, revealing potential noninvasive biomarkers (Leung and Wu, 2015; Matamala et al., 2018). Here, we conducted a systematic identification of circulating small RNAs differentially expressed in exosomes isolated from blood samples of Peruvian patients affected by latent tuberculosis, active tuberculosis, or pulmonary adenocarcinoma, when compared with healthy individuals. Literature reports that patients cured of tuberculosis represent a group at risk for developing pulmonary adenocarcinoma, probably because of the changes caused by the infectious disease in the pulmonary tissue (Cukic, 2017). Furthermore, it is known that macrophages infected with *Mycobacterium tuberculosis* can release different types of miRNAs packaged in exosomes into the extracellular space (Singh et al., 2015).

Our exosomal sRNA sequencing revealed a heterogeneous group of RNA classes differentially expressed in all studied conditions, with several of them exclusive for each disease, but with a high number of sRNAs shared between the different types of tuberculosis and pulmonary adenocarcinoma. In total, we found 24 miRNAs differentially expressed in at least one of the diseases, with only one miRNA shared within all conditions (miR-125a-5p). It was reported in literature that this miRNA may act as a tumor suppressor in various cancer types, such as breast (Yan et al., 2018) and colon (Tong et al., 2015; Yan et al., 2018), where presented expression levels were significantly reduced. When exosomes are released from the original cells, they fuse with the recipient cells and can act by controlling gene expression (Lv et al., 2020). In this context, a reduction of miR-125a-5p could decrease the gene control performed by this miRNA. Interestingly, miR-125a-5p was reported to be a direct regulator of the anti-apoptotic genes *BCL2*, *BCL2L12*, and *Mcl-1* in colon cancer cells in a negative manner (Tong et al., 2015). In this sense, miR-125a-5p presents an antitumoral effect in cells by direct negative expression regulation of genes suppressing apoptotic pathways. Its reduction in exosomes could be indicative of a decrease in general control that could favor the cancer progression. Additionally, during bacterial/viral infection it was reported that miR-125a-5p tends to be highly expressed and acts regulating known immunological responses of macrophages (Wang et al., 2020). Reports indicate that miR-125a-5p inhibits, in a post-transcriptional manner, the expression of *STAT3* (You et al., 2015; Wang et al., 2020), a transcription factor that has been observed to activate genes involved in the negative regulation of autophagy (*BCL2*, *BCL2L1*, *MCL1*, *PIK3R1/p55α*, and *PIK3R1/p50α*) and downregulate essential autophagy-related genes (*BECN1* and *PIK3C3*) (You et al., 2015; Wang et al., 2020). A decrease in the expression of miR-125a-5p suggests a reduction of the immune response for bacterial infection, such as *M. tuberculosis*. We suppose that *M. tuberculosis* uses this mechanism to evade or attenuate the host immune response in a way that the reduction of miR-125a-5p could be a key step to restrict autophagy. In this context, miR-125a-5p could be an important sRNA in normal tissues to maintain homoeostasis in pulmonary tissues, therefore its reduced expression in patients’ exosomes suggests its potential role as a putative biomarker for different states of tuberculosis or pulmonary adenocarcinoma.

Related to exclusive sRNAs, we found 3 miRNAs for LTB (miR-143-3p, miR-210-3p and miR-20a-5p), 4 for ATB (mir-23b, mir-17, mir-584 and miR-181b-5p), and only 1 shared exclusively within both types of tuberculosis (mir-203a). All these miRNAs appeared as downregulated, with the exception of miR-20a-5p (for LTB) and mir-584 (for ATB), which appeared as upregulated. These exclusive miRNAs have been reported to be involved in regulating cell proliferation (He et al., 2016; Luo et al., 2016; Yang et al., 2017) and therefore, are players for cellular homeostasis. However, the exclusive miRNAs that were upregulated (miR-20a-5p and mir-584) seem to favor mycobacterial infection through its post-transcriptional action. For example, the miR-20a-5p functioned as a negative regulator of mycobacterial-triggered apoptosis by expression regulation of the pro-apoptotic Bim protein, in a JNK2-dependent manner, promoting mycobacterial survival in infected THP-1 macrophages (Zhang et al., 2016). In this sense, related to LTB, the miR-20a-5p appears to be required to maintain the survival of mycobacterial infection.

In ATB, the mir-584 was found to be upregulated in our analysis. This miRNA has been associated with the inference of cell differentiation and tissue remodeling in *Helicobacter pylori* infection (Staedel and Darfeuille, 2013). There, the stem cell transcription factor Foxa1 is an important target of miRNA-584 and miRNA-1290, therefore the upregulation of mir-584 by CagA in a NF‐κB‐dependent manner repress Foxa1 and promotes epithelial to mesenchymal transition (Zhu et al., 2012). This could be similar to mycobacterial infection. Related to tuberculosis, recently the hsa-miR-584-5p has been predicted to have target genes related to *M. tuberculosis* infection, such as innate immune response, cell proliferation, and apoptosis (Han et al., 2020). The results showed that IL-6, a pleiotropic cytokine that plays a central role in host defense, is a target of hsa-miR-584-5p together with hsa-miR-484 and hsa-miR-4732-5p, suggesting it is an important player in host-pathogen interaction (Han et al., 2020). Therefore, increase of miR-584 could impact in the defense as mycobacterial infection, favoring their persistence such as in ATB.

In respect to other miRNAs, Alipoor et al. reported 3 miRNAs (miR-484, miR-425, and miR-96) with high expression in ATB as possible biomarkers (Alipoor et al., 2019). In another study, using pleural effusion samples, 3 other miRNAs (miR-148a-3p, miR-451a, and miR-150-5p) presented different expression patterns in TB patients from lung adenocarcinoma and other benign lesions (Wang et al., 2017). Interestingly, the use of differentially expressed miRNAs from each condition resulted in the identification of several metabolic pathways that could be altered by the dysregulation of these miRNA repertoire. Presented in the three conditions analyzed, the presence of paths related to cancer diseases suggest the importance of these miRNAs for the cellular homeostasis, suggesting indeed a link for the progression from LTB to a PA.

Altogether, our results are in accordance with other works, revealing the vastness and complexity of the circulating small RNA classes available in human exosomes and reinforcing their potential as noninvasive biomarkers for human diseases. It suggests a set of miRNAs that could be potential biomarkers for both types of tuberculosis (latent and active) and for pulmonary adenocarcinoma in the Peruvian population. Additional experimental procedures related to sensitivity and specificity are required to be performed in the future to select the specific miRNAs to be tested as biomarkers. Furthermore, our results indicate that there are some small RNAs, including micro RNAs, that are shared between LTB and ATB, suggesting that they could be associated with the transition between both types of tuberculosis, as well as the progression between tuberculosis and PA. Our findings constitute a valuable resource for the study of small RNAs as biomarkers for a specific population and is the first report focused on LTB, ATB, and PA specific to Peruvians.

## Data Availability Statement

The datasets presented in this study can be found in the SRA/NCBI repository with the bioproject accession number PRJNA644656.

## Ethics Statement

The studies involving human participants were reviewed and approved by Ethics Committees of the National Institute of Health and the National Institute of Cancer, Peru. The patients/participants provided their written informed consent to participate in this study.

## Author Contributions

MG, OP-C, SC, HLG, MO, and CS conducted the subjects sampling and molecular biology experiments. VA-T and VM-C conducted the bioinformatics analyses. HG, VA-T, and VM-C wrote and reviewed the manuscript. VM-C conceived and supervised the bioinformatics analyses. HG conceived and supervised the research. All authors read and approved the final manuscript.

## Funding

This work was funded in grants from Instituto Nacional de Salud (INS OI-038-15), FINCyT (OI-010-14) and FONDAP-ANID (15130011).

## Conflict of Interest

The authors declare that the research was conducted in the absence of any commercial or financial relationships that could be construed as a potential conflict of interest.

## Publisher’s Note

All claims expressed in this article are solely those of the authors and do not necessarily represent those of their affiliated organizations, or those of the publisher, the editors and the reviewers. Any product that may be evaluated in this article, or claim that may be made by its manufacturer, is not guaranteed or endorsed by the publisher.
