# Supplementary material for: Comparative Profiling of Circulating Exosomal Small RNAs Derived From Peruvian Patients With Tuberculosis and Pulmonary Adenocarcinoma
Source: Front Cell Infect Microbiol. 2022 Jun 30;12:909837. doi: 10.3389/fcimb.2022.909837 (PMC9280157; doi:10.3389/fcimb.2022.909837)
Supplement: Supplementary file 1 [file Table_1.docx]

**Supplementary Table 1.** Clinical characteristics of studied subjects.

| **Subject** | **Diagnosis** | **Gender** | **Age** | **BCG scar*** | **TST Test** | **TTF-1** |
| --- | --- | --- | --- | --- | --- | --- |
| 103000 | Healthy | F | 26 | Yes | 0 mm | - |
| 118000 | Healthy | F | 17 | No | 4 mm | - |
| 124000 | Healthy | F | 35 | Yes | 0 mm | - |
| 130000 | Healthy | M | 31 | Yes | 0 mm | - |
| 113000 | Latent Tuberculosis | F | 44 | Yes | 20 mm | - |
| 144000 | Latent Tuberculosis | F | 38 | Yes | 15 mm | - |
| 200000 | Latent Tuberculosis | M | 39 | Yes | 15 mm | - |
| 550000 | Latent Tuberculosis | M | 36 | Yes | 27 mm | - |
| 210000 | Active Tuberculosis | F | 18 | No | 14 mm | - |
| 374000 | Active Tuberculosis | F | 42 | Yes | 25 mm | - |
| 154000 | Active Tuberculosis | M | 46 | Yes | 26 mm | - |
| 196000 | Active Tuberculosis | M | 27 | Yes | 11 mm | - |
| 586330 | Pulmonary Adenocarcinoma | F | 67 | - | - | NA |
| 589802 | Pulmonary Adenocarcinoma | M | 54 | - | - | Positive |
| 589440** | Pulmonary Adenocarcinoma | M | 68 | - | - | Positive |
| 589943 | Pulmonary Adenocarcinoma | M | 71 | - | - | Positive |
| 589624** | Pulmonary Adenocarcinoma | M | 67 | - | - | Positive |
| 591923 | Pulmonary Adenocarcinoma | F | 61 | - | - | Positive |
| 585757** | Pulmonary Adenocarcinoma | F | 82 | - | - | Positive |

* Bacille Calmette-Guérin (BCG) vaccination scar. NA: not analyzed

** Not performed sRNA sequencing.
