# Supplementary material for: Comparative Profiling of Circulating Exosomal Small RNAs Derived From Peruvian Patients With Tuberculosis and Pulmonary Adenocarcinoma
Source: Front Cell Infect Microbiol. 2022 Jun 30;12:909837. doi: 10.3389/fcimb.2022.909837 (PMC9280157; doi:10.3389/fcimb.2022.909837)
Supplement: Supplementary file 3 [file Table_3.docx]

**Supplementary Table 3**. List of statistically differentially expressed circulating microRNAs in each studied disease. The arrows indicates if the miRNA is up (↑) or downregulated (↓) in each disease (log2 fold-change cutoff of 1.5; *p-value* cutoff of 0.05) compared to healthy controls, while the asterisks (*) indicates unperturbed miRNAs. The log2 fold-change and related p-value is indicated in parenthesis.

| **microRNA** | **LTB** | **ATB** | **PA** |
| --- | --- | --- | --- |
|  | *↑↓ \| **log2 fold-change \| *p-value*** | | |
| miR-125a-5p | ↓ \| -7.75 \| 0.006 | ↓ \| -5.57 \| 0.042 | ↓ \| -5.59 \| 0.031 |
| mir-203a | ↓ \| -8.08 \| 0.011 | ↓ \| -6.74 \| 0.035 | * \| -5.35 \| 0.067 |
| miR-143-3p | ↓ \| -4.33 \| 0.015 | * \| -1.09 \| 0.535 | * \| -2.20 \| 0.180 |
| miR-210-3p | ↓ \| -9.35 \| 0.029 | * \| -8.01 \| 0.062 | * \| -7.37 \| 0.062 |
| miR-20a-5p | ↑ \| 5.51 \| 0.041 | * \| 5.22 \| 0.054 | * \| 1.72 \| 0.522 |
| mir-23b | * \| -1.43 \| 0.494 | ↓ \| -6.13 \| 0.010 | * \| -1.55 \| 0.435 |
| mir-17 | * \| -1.70 \| 0.488 | ↓ \| -6.68 \| 0.014 | * \| -2.08 \| 0.367 |
| mir-584 | * \| 5.02 \| 0.067 | ↑ \| 6.35 \| 0.021 | * \| -0.19 \| 0.946 |
| miR-181b-5p | * \| -1.33 \| 0.570 | ↓ \| -5.26 \| 0.039 | * \| -4.02 \| 0.086 |
| mir-320a | * \| -2.09 \| 0.203 | * \| -1.92 \| 0.241 | ↓ \| -4.69 \| 0.002 |
| miR-185-5p | * \| -1.37 \| 0.507 | * \| -1.70 \| 0.413 | ↓ \| -6.39 \| 0.003 |
| miR-144-3p | * \| -0.66 \| 0.747 | * \| -0.48 \| 0.816 | ↓ \| -5.98 \| 0.005 |
| let-7f-5p | * \| -0.35 \| 0.781 | * \| 0.03 \| 0.984 | ↓ \| -2.82 \| 0.016 |
| miR-199b-3p | * \| -0.92 \| 0.540 | * \| -0.73 \| 0.630 | ↓ \| -3.57 \| 0.017 |
| let-7g-5p | * \| 0.05 \| 0.979 | * \| 0.96 \| 0.598 | ↓ \| -4.15 \| 0.020 |
| let-7i | * \| 0.14 \| 0.934 | * \| 0.23 \| 0.887 | ↓ \| -3.60 \| 0.021 |
| let-7f-1 | * \| -0.73 \| 0.577 | * \| 0.45 \| 0.729 | ↓ \| -2.81 \| 0.022 |
| miR-192-5p | * \| -2.47 \| 0.200 | * \| -1.27 \| 0.510 | ↓ \| -3.93 \| 0.034 |
| mir-101-2 | * \| -1.50 \| 0.432 | * \| -2.79 \| 0.148 | ↓ \| -3.80 \| 0.036 |
| mir-92a-1 | * \| -1.03 \| 0.518 | * \| -2.44 \| 0.127 | ↓ \| -3.10 \| 0.037 |
| mir-199a-1 | * \| -0.60 \| 0.676 | * \| -0.13 \| 0.927 | ↓ \| -2.88 \| 0.042 |
| miR-4508 | * \| -1.79 \| 0.457 | * \| -0.92 \| 0.706 | ↓ \| -4.91 \| 0.044 |
| let-7a-5p | * \| -0.18 \| 0.900 | * \| 0.42 \| 0.771 | ↓ \| -2.67 \| 0.048 |
| miR-181a-5p | * \| -0.12 \| 0.926 | * \| -0.82 \| 0.532 | ↓ \| -2.45 \| 0.049 |
